# Beyond Palmer: a topographic classification of non-Palmer triangular fibrocartilage complex injuries

**DOI:** 10.1186/s13244-026-02356-8

**Published:** 2026-08-06

**Authors:** Alvaro Cerezal, Francisco del Piñal, Alejandro Rolón, Ana Canga, Alexeys Perez, Luis Cerezal

**Affiliations:** 1 Department of Orthopedic Surgery, La Paz University Hospital, Madrid, Spain; 2 Institute of Reconstructive Traumatology and Hand Surgery Piñal & Associates, Madrid, Spain; 3 Department of Radiology, Centro Diagnóstico Himan, Buenos Aires, Argentina; 4 Department of Anatomy, Cantabria University. Department of Radiology, Valdecilla University Hospital, IDIVAL, Santander, Spain; 5 Department of Radiology, Iberorad, Barcelona, Spain; 6 Diagnóstico Médico Cantabria (DMC), Santander, Spain

**Keywords:** Wrist, Wrist injuries, Triangular fibrocartilage, Magnetic resonance imaging, Arthroscopy

## Abstract

**Abstract:**

Triangular fibrocartilage complex (TFCC) injuries are a common cause of ulnar-sided wrist pain. The Palmer classification remains the standard framework, but high-resolution MRI and wrist arthroscopy have revealed injury patterns not included in the original scheme. These include refinements of foveal avulsions (Palmer type 1B) and radial-sided tears (type 1D), as well as broader anatomical and functional concepts of TFCC organization. In this educational review, we present a topographic classification of non-Palmer TFCC injuries into three groups: central lesions, capsular tears, and complex or combined injuries. Each group has different therapeutic implications. We review the relevant pathomechanics, clinical presentation, and imaging findings, with emphasis on MRI and MR or CT arthrography, and correlate them with arthroscopic and surgical findings to guide treatment planning.

**Key Points:**

- **Question:** although the Palmer classification remains the standard framework for TFCC injuries, MRI and arthroscopy frequently demonstrate clinically relevant lesion patterns beyond its original categories.
- **Findings:** non-Palmer TFCC injuries fall into three topographic categories: central, capsular, and complex or combined lesions, each with characteristic MRI features and treatment implications.
- **Critical relevance statement:** topographic classification of non-Palmer TFCC injuries supports more accurate preoperative MRI interpretation and surgical planning in patients with ulnar-sided wrist pain.

**Graphical Abstract:**

**Figure:**
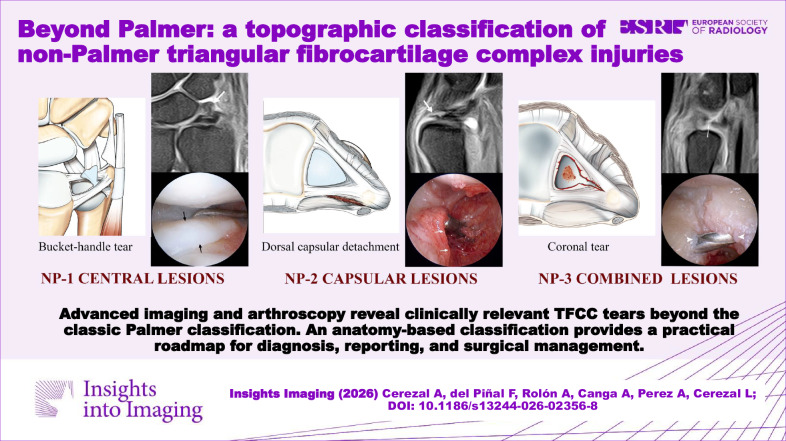

## Introduction

The triangular fibrocartilage complex (TFCC) is the primary static stabilizer of the distal radioulnar joint (DRUJ) and the ulnocarpal unit [1–3]. Ulnar-sided wrist pain remains one of the more demanding diagnostic problems in musculoskeletal imaging: pathologies frequently overlap, and clinical examination tests offer limited discriminatory accuracy, often leaving the assessment inconclusive. In this context, high-resolution imaging is essential for establishing a diagnosis and ruling out competing causes [4–6].

Since its introduction in 1989, the Palmer classification has been the accepted classification system for TFCC pathology, establishing a common language for radiologists and hand surgeons [1]. This classification scheme was originally developed from a radiocarpal arthroscopic perspective and represents a two-dimensional topographic approach. As a result, it does not fully capture the three-dimensional architecture of the TFCC, tends to group lesions with substantially different biomechanical consequences under a single label, and falls short of reliably predicting DRUJ or ulnocarpal instability, arguably the most consequential determinants of both prognosis and surgical strategy [4, 5]. These shortcomings become most apparent in foveal attachment injuries, the so-called “iceberg” lesions, where clinically significant tears along the proximal (DRUJ-facing) surface may go undetected on standard radiocarpal inspection [4].

Over the past two decades, advances in wrist arthroscopy and high-resolution magnetic resonance imaging (MRI), further supported by direct intra-articular contrast techniques such as MR arthrography and computed tomography (CT) arthrography, have expanded the recognized spectrum of TFCC injury beyond the classic Palmer categories [6, 7]. Several refinements and alternative classification models have since been proposed to address these gaps; yet their integration into routine clinical practice has remained uneven [8–10].

This educational review uses the Palmer classification as its baseline, incorporating its most clinically relevant refinements while organizing the remaining injury configurations into the broader category of “non-Palmer” TFCC lesions within a unified topographic framework. Throughout, the relevant functional anatomy, a structured imaging strategy, and the arthroscopic correlates of each lesion are discussed to guide preoperative planning and surgical management. This work draws on the authors’ experience and published research in musculoskeletal radiology, wrist arthroscopy, and hand surgery [7].

## Anatomy of the TFCC

**Fig. 1:**
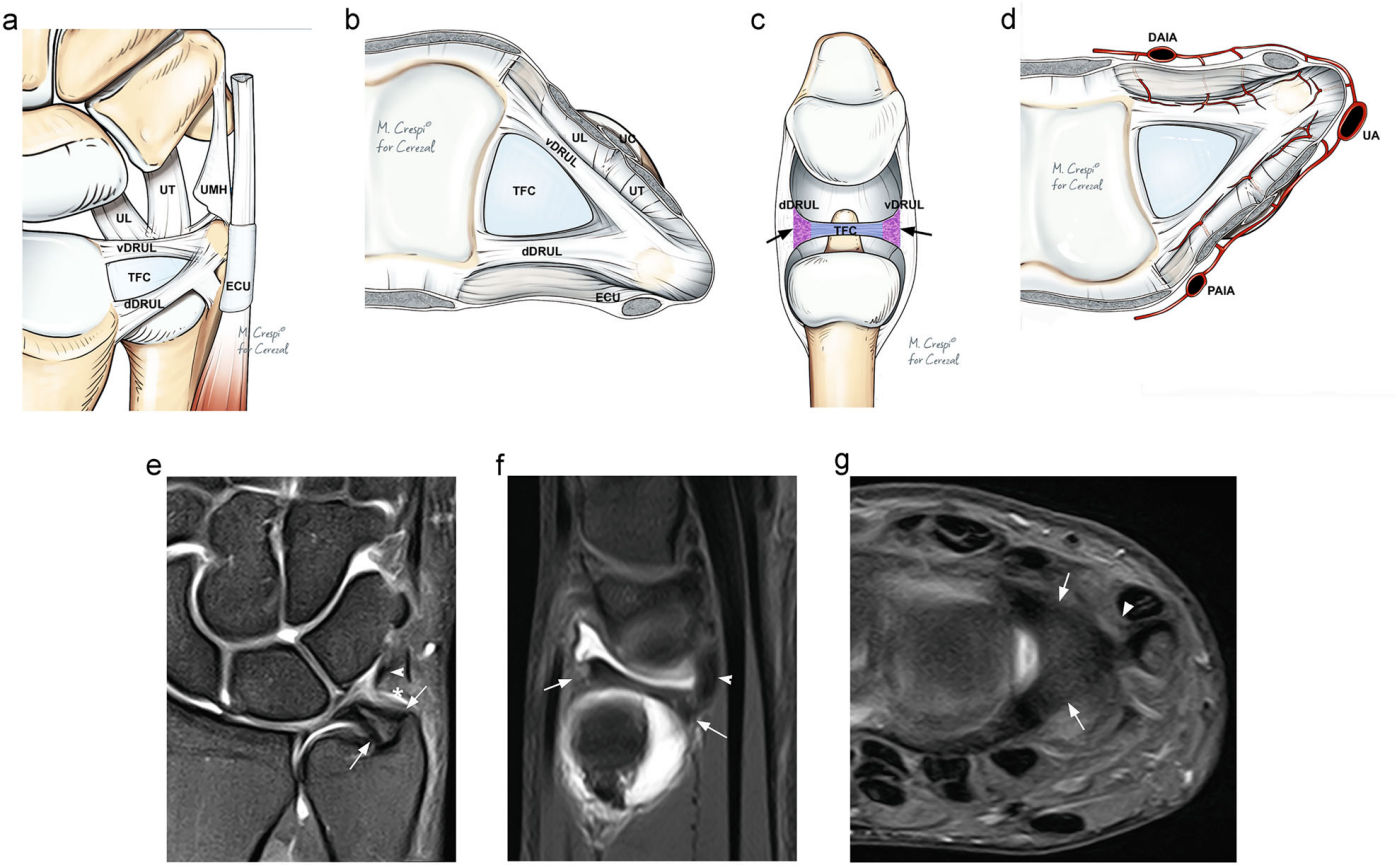
Normal TFCC anatomy. **a** Posteroanterior and (**b**) axial diagrams. The TFCC comprises the triangular fibrocartilage (TFC) proper, dorsal and volar DRULs, ulnocarpal ligaments (UL, UT, and ulnocapitate [UC]), UMH, and the ECU subsheath. **c** Sagittal diagram of the TFC proper and DRUL with capsular attachments (arrows). **d** Vascular supply: the ulnar artery (UA) and palmar (PAIA) and dorsal (DAIA) branches of the anterior interosseous artery perfuse the peripheral TFCC; the central TFC proper is avascular. **e** Coronal proton-density fat-suppressed MRI demonstrates the radial and ulnar insertions, including styloid and foveal laminae (arrows), the UMH (arrowhead), and the prestyloid recess (asterisk). **f** Sagittal proton-density fat-suppressed MRI depicts the TFC proper, volar, and dorsal DRUL with capsular attachments (arrows) and the UT ligament (arrowhead). **g** Axial proton-density fat-suppressed MRI delineates the DRUL with its capsular attachment (arrows) and the ECU subsheath (arrowhead)

The TFCC is a composite ligamentous and fibrocartilaginous structure located on the ulnar side of the wrist. It consists of the articular disc, the volar and dorsal distal radioulnar ligaments (DRULs), the ulnocarpal ligaments (ulnolunate (UL), ulnotriquetral (UT), and ulnocapitate), the ulnomeniscal homologue (UMH), the extensor carpi ulnaris (ECU) subsheath, and the ulnocarpal capsule (Fig. 1) [1, 11–13].

### Central articular disc (TFC proper)

The central articular disc, or triangular fibrocartilage (TFC) proper, is a biconcave fibrocartilaginous structure with a broad radial base that narrows toward the ulnar apex [13–15]. Its radial attachment anchors to the distal margin of the sigmoid notch by Sharpey fibers within a dense osteofibrous insertion [16]. At its volar and dorsal margins and at the ulnar apex, the TFC proper blends with the DRULs to form a mechanically integrated unit [15, 16].

### DRULs

The DRULs are the primary stabilizers of the DRUJ [2, 17]. Each consists of superficial and deep laminae. The resulting four discrete bundles originate along the margins of the radial sigmoid notch [18–22]. The superficial laminae follow a predominantly horizontal course toward the ulnar styloid. The deep laminae, or ligamentum subcruentum, run obliquely to the ulnar fovea via Sharpey fibers [18–21]. At this foveal insertion, the distal radioulnar and ulnocarpal ligament systems converge to establish the isometric center of forearm rotation and the principal anchor for DRUJ and ulnocarpal stability [16, 23]. During pronation-supination, the four bundles tighten reciprocally and maintain joint stability across the full rotational arc [2, 15, 21, 24]. Articular contact peaks in the neutral position and diminishes at the extremes of rotation. During pronation, the radius moves volarly, and the ulnar head displaces dorsally; in supination, the radius shifts dorsally, and the ulnar head moves volarly [21, 24, 25].

### Ulnocarpal ligament complex

The ulnocarpal ligament complex comprises the UL, UT, and ulnocapitate (UC) ligaments [26]. All three arise from the ulnar fovea and ulnar styloid process and fan distally toward the lunate, triquetrum, and capitate to form a volar hammock that stabilizes the ulnocarpal joint and provides secondary DRUJ support [3, 15]. This complex restrains palmar carpal translation and limits dorsal displacement of the ulnar head [27]. The UT tightens maximally in radial deviation, the Ulnocapitate ligament (UC) in radial extension under axial load, and the UL in ulnar deviation and full wrist extension. In maximal supination, forearm rotation tensions the complex at its foveal and styloid origin. This tension coapts the ulnar head against the sigmoid notch [26].

### UMH

**Fig. 2:**
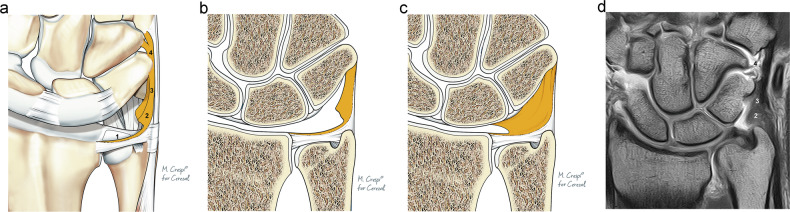
UMH anatomy and variants. **a** Coronal schematic drawing depicts the UMH and its distal radioulnar (1), styloid (2), collateral (3), and distal (4) portions, and its relationship to the deep margin of the ECU tendon. **b**, **c** Diagrams illustrate the open and closed UMH variants based on their triquetral insertion: in the open type (~ 90%), the UMH has a focal dorsoulnar attachment to the triquetrum; in the closed type (~ 10%), the attachment is broader and may extend to the lunotriquetral ligament. **d** Coronal fat-suppressed T1-weighted MR arthrography delineates the styloid (2), collateral (3), and distal (4) UMH components and their relationship with the ECU

The UMH is a peripheral component of the TFCC composed of longitudinal collagen fibers embedded in vascularized loose connective tissue and lined by synovium [28]. Anatomic studies have described four related regions within the UMH: the radioulnar, styloid, collateral, and distal insertional portions (Fig. 2) [28]. At the posterior margin of the sigmoid notch, the radioulnar portion is continuous with the dorsal DRUL and then blends into the styloid portion, which constitutes the main triangular segment distal to the articular disc. Its vascularized base is attached to the ECU sheath and ulnar styloid, whereas the collateral portion courses distally toward the radial margin of the ECU sheath [28].

Distally, the UMH attaches most consistently to the dorsoulnar aspect of the triquetrum, although additional variable insertions to the ulnar hamate, the base of the fifth metacarpal, and the dorsal pisotriquetral capsule have also been described [28]. Two patterns of triquetral insertion have been reported [12]: a focal dorsoulnar attachment (“open” type), present in approximately 90% of specimens, and a broader dorsal attachment (“closed” type), seen in approximately 10%, which may extend to the lunotriquetral ligament. The prestyloid recess, a synovial space located between the disc apex and the UMH, is variable in size and may be absent [12]. Functionally, the UMH acts as a compliant soft-tissue buffer along the ulnar carpus. Its hammock-like configuration becomes lax and folds during ulnar deviation, whereas it tightens during radial deviation [21, 29].

### ECU subsheath (infratendinous extensor retinaculum)

The ECU subsheath, or infratendinous extensor retinaculum, is an important dorsal stabilizer of both the DRUJ and the ECU tendon [30, 31]. Together with the dorsal ulnar groove, it forms the osteofibrous canal through which the tendon passes during forearm rotation. The subsheath measures approximately 15–20 mm in length and lies deep to the superficial extensor retinaculum, which overlies the ECU tendon sheath and inserts distally onto the hamate and pisiform without direct ulnar attachment [31].

Proximally, the subsheath is continuous with the dorsal DRUL and attaches to the ulnar fovea and styloid through Sharpey fibers. Distally, it extends toward the dorsal triquetrum, linking the TFCC to the dorsal capsule [31]. Three histologic regions have been described: a thin elastic segment within the ulnar groove, a thicker styloid portion continuous with the dorsal DRUL, and a distal triquetral portion that helps maintain tendon containment [31]. Functionally, the subsheath keeps the ECU tendon centered during forearm rotation. It becomes taut in pronation, helping resist dorsal displacement of the ulna, and relaxes in supination, when a small degree of physiologic tendon subluxation may be present [30, 31].

### Vascularity

The central and radial portions of the TFC proper are predominantly avascular. The peripheral zone comprises 10–40% of the disc by area and receives a rich supply from branches of the ulnar and anterior interosseous arteries [32]. This vascular gradient determines both prognosis and treatment. Peripheral lesions within the well-vascularized zone have intrinsic healing potential and are amenable to primary repair. Central avascular tears are generally managed with debridement [33–35].

### Innervation

The TFCC is innervated by multiple neural sources [36, 37]. The dorsal cutaneous branch of the ulnar nerve is the most consistent source. It supplies the medial and posterior aspects. The palmar cutaneous branch and the medial antebrachial cutaneous nerve cover the anteromedial portion. The posterior interosseous nerve provides variable contributions to the dorsal region [36]. Immunohistochemical studies confirm fine sensory fiber ingrowth throughout the complex. Nerve fiber density is highest in the dorsal segment [37]. This distribution accounts for the recalcitrant ulnar-sided wrist pain seen after peripheral TFCC tears.

### Functional anatomy of the TFCC

Nakamura proposed a functional subdivision of the TFCC into three integrated components [23]. The distal ulnocarpal support unit comprises the TFC proper, UMH, and ulnocarpal ligaments. This unit suspends the ulnar carpus and undergoes minimal intrinsic deformation during pronation-supination. The proximal stabilizing unit is formed by the DRULs. It provides primary DRUJ stabilization throughout the rotational arc. The functional ulnar collateral ligament is formed by the ECU subsheath and a thickening of the ulnar capsule. It translates dorsally in pronation and palmarly in supination, contributing to rotational DRUJ control [15, 21, 23].

## Imaging strategy for suspected non-Palmer (NP) TFCC injuries

### Ultrasound

Conventional radiography remains the initial imaging study, but in everyday practice, ultrasound has become a practical first-line tool for assessing acute and chronic ulnar-sided wrist pain [38]. Its diagnostic performance for intrinsic TFCC tears is limited; however, it remains valuable in other respects [7]. High-frequency hockey-stick transducers (17–22 MHz) provide sufficient spatial resolution to evaluate peripheral structures, including the ulnar styloid and foveal attachments, the dorsal capsule, and the ECU subsheath, particularly when the wrist is examined in neutral rotation or forced flexion [39, 40]. Color and power Doppler are useful for detecting synovial thickening, active synovitis, and pericapsular inflammation. Dynamic examination, ideally compared with the contralateral side, is particularly helpful for assessing ECU instability and abnormal DRUJ motion, as these findings are best appreciated in real time rather than on static imaging [39, 40]. Ultrasound may also depict the dorsal distal radioulnar tract of the interosseous membrane, which can be relevant in dorsal capsular injury patterns. By contrast, the deep TFCC components cannot be assessed reliably with ultrasound [7].

### Computed tomography

Multidetector CT with isotropic acquisition is the reference technique for evaluating the osseous anatomy of the DRUJ, including fractures, malunion, and rotational malalignment [25]. For the assessment of instability, static acquisitions in neutral position, maximal pronation, and maximal supination are commonly included in the protocol. Four-dimensional CT is an emerging technique that adds kinematic information, and loading maneuvers performed during image acquisition may reveal subtle displacement not evident on static studies [25].

### Magnetic resonance imaging

MRI is the main imaging modality for assessing TFCC soft-tissue integrity, DRUJ alignment, and indirect signs of instability, and it plays a central role in the differential diagnosis of ulnar-sided wrist pain [41–46]. Optimal evaluation requires high-field-strength systems (1.5–3 T), dedicated wrist coils, and thin-section small-field-of-view protocols. Fat-suppressed proton density-weighted sequences in the three orthogonal planes form the basis of the examination, whereas isotropic three-dimensional acquisitions allow multiplanar reformatting along individual TFCC components [47, 48]. Although not routinely used in daily practice, contrast-enhanced fat-saturated T1-weighted sequences may improve the detection of foveal and styloid TFCC injuries [49].

Accurate evaluation of the TFCC requires a systematic multiplanar approach. Coronal images are best suited for assessment of the articular disc and radioulnar ligament insertions. Sagittal images are particularly helpful for assessing the capsular attachments and the UMH, especially at the dorsal triquetrum. Axial images are essential for evaluating the volar and dorsal capsular insertions, the ECU subsheath, and DRUJ alignment [21]. Precise plane orientation is critical because even minor deviations may alter the appearance of the ulnar-sided structures and lead to false-positive or false-negative interpretation of peripheral detachments [21, 50]. Axial images obtained in neutral rotation may show static malalignment, whereas additional acquisitions in pronation and supination can help demonstrate instability that is not apparent in the neutral position [15, 21].

### Arthrographic techniques

MR arthrography and CT arthrography are indicated when clinical suspicion remains high despite negative or inconclusive conventional MRI findings [43, 50, 51]. Their main indications include partial or non-communicating tears of the ulnar attachment, particularly foveal tears, suspected NP injury patterns, and selected degenerative cases requiring detailed evaluation of the lunotriquetral region [7]. Baseline MRI should be performed before arthrography, because intra-articular contrast may obscure indirect inflammatory findings, such as synovitis and pericapsular edema, that can otherwise assist in diagnosis [21, 50, 51].

Capsular distension during arthrography may open cleavage planes that are not visible on routine MRI, thereby improving detection of dorsal and volar capsular detachments, partial type 1B tears, and proximal delamination [21]. The injected compartment is important. DRUJ injection is required to demonstrate non-communicating foveal or central flap tears and lesions of the proximal TFCC surface, whereas radiocarpal injection provides better opacification of styloid-sided tears and peripheral injury patterns such as the carpal detachment described by Nishikawa [21, 50]. Radial multiplanar reformations in both MR and CT arthrography improve diagnostic accuracy for peripheral TFCC detachments and foveal tears [52–54]. Of the two techniques, MR arthrography is generally preferred because of its superior soft-tissue contrast, whereas CT arthrography is mainly reserved for detailed osseous assessment or for patients in whom MRI is contraindicated [15, 50].

### Arthroscopy

Wrist arthroscopy remains the reference standard for both diagnosis and treatment of TFCC pathology and has been instrumental in defining injury patterns that are not included in the Palmer classification, such as dorsal and volar capsular detachments, delaminating flap tears, bucket-handle tears, carpal detachment (Nishikawa lesion), and complex combined lesions [9, 10, 18].

Standard evaluation through radiocarpal portals, typically with 3–4 viewing and 6 R instrumentation, allows inspection of the distal TFCC surface and dynamic assessment using the trampoline and hook tests [4, 55]. However, the proximal DRUJ-facing surface cannot be visualized from the radiocarpal side. This limitation, often referred to as the “iceberg” phenomenon, means that an apparently normal radiocarpal arthroscopic appearance may coexist with foveal avulsion, proximal tearing, or substantial delamination on the opposite surface [4, 55, 56]. Preoperative imaging is therefore essential to identify cases in which DRUJ arthroscopy should be performed. Although technically demanding, DRUJ arthroscopy is the only method that allows direct assessment of the foveal fibers [57, 58]. Accurate preoperative lesion localization and careful correlation between imaging and arthroscopic findings are critical for appropriate treatment and restoration of stability.

## Evolution of TFCC injury classification

### Limitations of the Palmer classification

The Palmer classification, introduced in 1989, addressed a clear need by providing radiologists and hand surgeons with a common framework for describing TFCC injuries. The system separates TFCC lesions into traumatic (class 1) and degenerative (class 2) categories (Table 1) [1]. Traumatic tears are further subdivided into four topographic patterns (1A–1D), whereas degenerative lesions are arranged as a progressive spectrum related to ulnocarpal impaction, ranging from central disc wear to ulnocarpal osteoarthritis (2A–2E) [1]. Its main strengths are simplicity and reproducibility.

**Table 1:** Palmer classification of TFCC lesions

Palmer class 1: Traumatic	
1A	Central TFC slit
1B	Ulnar avulsion with or without ulnar styloid fracture
1C	Distal avulsion of ulnocarpal ligaments (carpal attachment)
1D	Radial avulsion with or without sigmoid notch fracture
Palmer class 2: degenerative	
2A	TFCC wear
2B	TFCC wear, lunate and/or ulnar chondromalacia
2C	TFCC perforation, lunate and/or ulnar chondromalacia
2D	TFCC perforation, lunate and ulnar chondromalacia, and lunotriquetral ligament tear
2E	Ulnocarpal arthrosis

Subsequent advances in wrist arthroscopy and high-resolution MRI have also made its limitations increasingly apparent [59–61]. The Palmer classification is based primarily on radiocarpal arthroscopy and reflects a largely two-dimensional topographic interpretation of a complex three-dimensional structure. As a result, lesions with very different biomechanical and therapeutic implications may be grouped under the same category. More importantly, the classification does not reliably predict DRUJ or ulnocarpal instability [4–7]. These shortcomings have led to refinements focused on stability and reparability, as well as to the development of complementary classification models that address aspects not captured by the original Palmer system [4–10].

### Refinements of the Palmer classification

Subsequent proposals have not sought to replace the Palmer classification, but rather to refine those categories in which treatment depends on factors not adequately reflected in the original system.

#### Peripheral ulnar-sided tears (type 1B)

Type 1B is widely regarded as the most important limitation of the original classification. All peripheral ulnar-sided tears are grouped under the same label, regardless of whether the DRUJ remains stable or whether the lesion is suitable for repair. Atzei and Luchetti described this limitation using the “iceberg” concept: radiocarpal arthroscopy evaluates only the distal surface of the TFCC, whereas the foveal insertion, the principal stabilizing attachment of the DRUJ, is not directly visible. A lesion that appears limited from the radiocarpal side may therefore be associated with proximal detachment or foveal avulsion of much greater clinical significance [61, 62].

Atzei subclassified Palmer class 1B lesions using criteria relevant to treatment rather than topography alone: foveal integrity, DRUJ stability, and tissue reparability [4, 62, 63]. The scheme defines five classes, each tied to a specific surgical decision. Class 1 tears are superficial and styloid-sided. The fovea is intact, and the DRUJ is stable, and arthroscopic capsular or ECU subsheath suture restores the torn component. Class 2 tears are complete ulnar detachments. Both the distal and proximal components are disrupted, the DRUJ is unstable, and foveal refixation is required. Class 3 is an isolated, deep-foveal avulsion. Because the superficial component remains intact, the lesion is frequently occult at radiocarpal arthroscopy; the DRUJ is nonetheless unstable, and the fovea must be reattached with transosseous sutures or suture anchors. Repair is no longer an option in the last two classes. Class 4 tears are chronic and irreparable, with poor tissue quality or large defects that make primary repair futile; tendon graft reconstruction of the DRUL is needed instead. Class 5 applies when DRUJ arthritis is already established. Repair is contraindicated, and salvage procedures such as resection, arthroplasty, or joint replacement are indicated [4, 62–64].

Del Piñal’s concept of the “1B constellation” adds a related perspective [5]. In many cases, peripheral TFCC failure does not involve a single isolated structure. Instead, ulnar-sided wrist pain and instability may result from combined injury to the styloid and foveal insertions, the capsule, the ECU subsheath, and, in some cases, an associated fracture of the ulnar styloid. In this context, the 1B label should be regarded as an initial category rather than a complete diagnosis [5].

#### Radial-sided tears (type 1D)

Palmer type 1D tears were originally defined as complete avulsions of the TFCC from its radial attachment [1]. Nakamura subsequently subclassified radial-sided tears into five types based on arthroscopic findings [65], a scheme later adapted to high-resolution MR imaging by Zhan [45]. Type (a) is a radial slit or flap tear confined to the fibrocartilaginous disc, without DRUJ instability. It is the most commonly identified pattern at radiocarpal arthroscopy and is treated with arthroscopic debridement. Type (b) is a dorsal rim tear, with or without an avulsion fracture of the dorsal margin of the sigmoid notch. Type (c) is a palmar-radial avulsion tear, with or without an avulsion fracture of the palmar margin of the sigmoid notch. Type (d) combines a radial disc tear with dorsal avulsion of the TFCC from the sigmoid notch. Type (e) is a total avulsion of the TFCC from the radius. Tears that involve the DRUL result in DRUJ instability and require radial reattachment with suture anchors [65].

The “pre-1D” concept introduced by Luchetti [66] added a further critical point: a mid-substance rupture of the radioulnar ligaments occurring approximately 5–10 mm proximal to their insertion on the sigmoid notch. This anatomic nuance dictates that apparent continuity of the radial footprint on imaging does not necessarily indicate that the attached stabilizing fibers remain functionally intact. Therapeutically, because the ligamentous footprint remains attached to the bone, this specific pattern is not amenable to the standard transosseous repair classically proposed for true Palmer 1D avulsions; instead, it mandates an alternative soft-tissue-to-soft-tissue suture technique [66].

#### MRI-based refinements of the Palmer classification

Alongside arthroscopic refinements, high-resolution MRI and imaging-arthroscopy correlation have documented injury patterns that do not fit within the original Palmer scheme, including horizontal intradiscal (delaminating) tears, peripheral capsular detachments, and bucket-handle configurations [4–10]. Zhan’s modified Palmer scheme attempted to refine the Palmer classification by incorporating these injury patterns [6]. Contemporary series consistently show that combined injury patterns are common and that reducing these lesions to a single dominant label almost certainly underestimates their frequency [5–8].

## New anatomic-functional classification systems

To address these shortcomings, several anatomic-functional classification systems have been proposed. These models move beyond simple topographic labeling and focus on which TFCC subcomponent is involved and how the injury affects stability and load transmission [8–10]. Schmitt’s CUP classification (Central-Ulnar-Peripheral) divides the TFCC into reproducible regions and grades lesion severity within each region, which allows combined injuries to be systematically described and aligns imaging findings with treatment strategy [8]. Herzberg’s three-dimensional D-R-W model conceptualizes the TFCC as a functional box composed of the Disc, the radioulnar ligament “Reins,” and the peripheral capsular “Wall”; each element corresponds to a specific biomechanical role: shock absorption, DRUJ stabilization, or ulnocarpal containment [9]. In contrast to these imaging-oriented schemes, Nakamura’s three-dimensional arthroscopic classification assesses both the distal and proximal TFCC surfaces, distinguishing between lesions that are painful but mechanically stable and those associated with true DRUJ instability [10].

These newer systems address several limitations of the Palmer classification, describing TFCC anatomy and biomechanics in greater detail. Their role in routine radiological reporting is still being defined. Because they are recent and more complex, they require broader dissemination and familiarization before their practical value can be judged. The Palmer classification, therefore, remains widely used because it is simple to apply, although it does not capture the full spectrum of TFCC pathology.

## A practical approach: Palmer as the baseline and NP injuries as a complementary classification

A practical approach is to use the Palmer classification as the basic framework. When relevant, apply the Atzei 1B and Nakamura 1D refinements, and group the remaining patterns under the broader category of NP lesions [7, 21]. Organizing NP lesions into central, capsular, and complex or combined patterns creates an anatomically coherent system (Table 2). This system is feasible for routine reporting and useful for treatment planning (Fig. S1) [7, 21]. The NP category also bridges imaging and arthroscopy. It provides a common descriptive vocabulary for lesion patterns that the Palmer subtypes do not adequately capture (Table 3) [21, 67].

**Table 2:** NP injuries classification

NP type	Category	Subtype	Lesion pattern
NP-1	Central lesions	NP-1A	Horizontal tear
		NP-1B	Delaminating (“flap”) or bucket-handle tears
NP-2	Capsular lesions	NP-2A	Volar capsular detachment
		NP-2B	Dorsal capsular detachment
		NP-2C	Injuries of the volar ulnocarpal ligaments
		NP-2D	Nishikawa lesion (carpal detachment)
		NP-2E	TILT syndrome (triquetral impingement ligament tear)
NP-3	Complex or combined lesions	NP-3A	Combined forms of traumatic injuries
		NP-3B	Combined traumatic and degenerative lesions

**Table 3:** Imaging-based reporting framework for NP TFCC lesions

NP subtype	Key imaging findings	Proposed report statement
NP-1—central lesions		
NP-1A—horizontal tear	Linear T2-hyperintense intrasubstance band parallel to the articular disc surface, with or without surface communication; possible splitting of the disc into two laminae. An adjacent ganglion or synovial cyst may be present. Purely intrasubstance tears may not opacify at arthrography.	Central NP lesion (NP-1A): horizontal intrasubstance cleavage tear of the articular disc, separating it into two laminae, with or without an adjacent ganglion/synovial cyst.
NP-1B—delaminating (“flap”) or bucket-handle tear	Unstable delaminating flap or folded bucket-handle fragment. A low-signal displaced fragment outlined by fluid may produce a “comma sign.” The fragment may extend into the radiocarpal compartment, the DRUJ, or both; occasionally, a free migrated fragment may be present.	Central NP lesion (NP-1B): delaminating flap/bucket-handle tear with [no displacement/displacement into the radiocarpal compartment and/or DRUJ], with or without a free migrated fragment.
NP-2—capsular lesions		
NP-2A—volar capsular detachment	Fluid-signal cleft between the volar TFCC margin and the palmar capsule on axial and sagittal images; possible separation of the volar DRUL component, often associated with focal synovitis and/or pericapsular edema.	Capsular NP lesion (NP-2A): volar capsular detachment, with [intact/abnormal] foveal attachment.
NP-2B—dorsal capsular detachment (including PARC)	Fluid-signal cleft between the dorsal TFCC margin and the dorsal capsule, often associated with focal synovitis and/or pericapsular edema. Assess for concomitant ECU subsheath injury and foveal tear. PARC refers to proximal avulsion of the radiocarpal capsule from the dorsal radial rim.	Capsular NP lesion (NP-2B): dorsal capsular detachment [with/without PARC involvement], with [intact/abnormal] foveal attachment and [intact/abnormal] ECU subsheath.
NP-2C—injuries of the volar ulnocarpal ligaments	Longitudinal intraligamentous split of the UT; MRI sensitivity is low; it mainly helps exclude alternative causes.	Capsular NP lesion (NP-2C): suspected longitudinal UT split.
NP-2D—Nishikawa lesion (carpal detachment)	Detachment of the dorsoulnar ulnocarpal capsule and UMH from the dorsal triquetral margin, with focal dorsoulnar synovitis/fibrosis and dorsal triquetral chondral delamination.	Capsular NP lesion (NP-2D): Nishikawa lesion, with detachment of the dorsoulnar ulnocarpal capsule and UMH from the dorsal triquetral margin, with [present/absent] dorsal triquetral chondral defect.
NP-2E—TILT syndrome (TILT)	Distally migrated fibrous cuff from the ulnar sling mechanism causing impingement on the ulnar margin of the triquetrum, with focal synovitis and triquetral chondropathy. Marrow edema, reactive sclerosis, or subchondral cysts may indicate chronic chondro-osseous degeneration.	Capsular NP lesion (NP-2E): TILT pattern, with fibrous cuff impingement on the triquetrum and [present/absent] triquetral chondro-osseous degeneration.
NP-3—complex or combined lesions		
NP-3A—combined forms of traumatic injuries	Combination of more than one traumatic TFCC injury component, such as central disc tear (Palmer 1 A) with peripheral ulnar-sided tear (Palmer 1B), dorsal capsular detachment with foveal involvement, or combined radial detachment (Palmer 1D) and ulnar detachment (Palmer 1B), producing a floating TFCC pattern.	Complex NP lesion (NP-3A): combined traumatic TFCC injury, with [specify components] and suspected instability driven by [foveal/radial / combined] involvement.
NP-3B—combined traumatic and degenerative lesions	Acute tear superimposed on a degenerated articular disc. Conventional MRI may underestimate lesion extent; MR or CT arthrography helps define the full extent of injury.	Complex NP lesion (NP-3B): acute-on-degenerative TFCC injury, with [specified acute and degenerative components] and suspected instability driven by [foveal/radial / combined] involvement.

*DRUJ* distal radioulnar joint, *DRUL* distal radioulnar ligament, *ECU* extensor carpi ulnaris, *PARC* proximal avulsion of the radiocarpal capsule, *TILT* triquetral impingement ligament tear

## NP injuries

### Central NP TFCC injuries (NP-1)

Two central injury patterns sit outside the Palmer classification: intrasubstance horizontal (cleavage) tears, delaminating tears with unstable flaps, or bucket-handle tears [7, 21].

#### Horizontal tears (NP-1A)

**Fig. 3:**
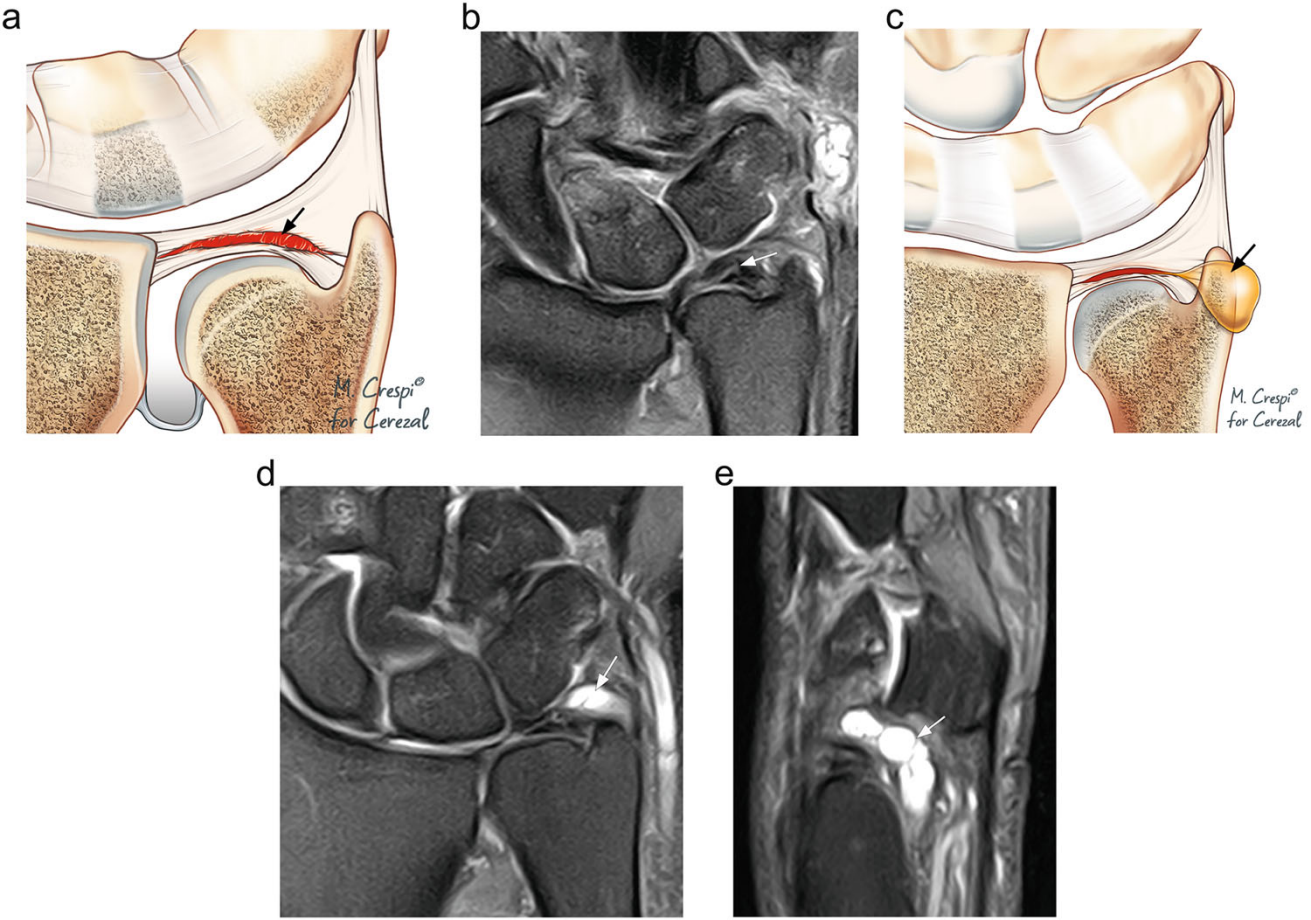
Horizontal tear of the TFC proper (NP-1A). **a** Coronal schematic drawing and (**b**) coronal proton-density fat-suppressed MRI demonstrate a horizontal cleavage tear of the TFC proper (arrows). **c** The coronal diagram depicts a horizontal tear with a secondary ulnar-sided synovial cyst (arrow). **d**, **e** Coronal and sagittal proton-density fat-suppressed MRI reveal a horizontal tear communicating with an ulnar-sided synovial cyst (arrows)

Horizontal tears (NP-1A) cleave the fibrocartilage into superior and inferior laminae, similar to the cleavage pattern seen in horizontal meniscal tears of the knee (Fig. 3) [5, 60]. Most reflect degenerative central disc change, often in the setting of ulnocarpal impaction, although acute trauma may also be responsible in some cases. Concomitant findings include central thinning, fibrillation, or perforation [21, 60]. The cleavage plane may function as a one-way valve, allowing synovial fluid to dissect peripherally and collect at the ulnar TFCC margin as a ganglion or synovial cyst [21]. On MRI, a cyst adjacent to the TFCC should suggest the possibility of an underlying horizontal tear. Patients may be asymptomatic or may report mechanical symptoms, such as clicking or intermittent locking, when laminar instability is present [5, 21].

On MRI, horizontal tears appear as linear T2-hyperintense intrasubstance bands running parallel to the disc surface and separating it into two laminae without surface communication [6, 21, 45]. Delamination may extend peripherally and coexist with adjacent cysts. Purely intrasubstance tears may not opacify on MR or CT arthrography unless small surface defects allow contrast entry, which limits arthrographic sensitivity for this pattern [21]. At arthroscopy, intact articular surfaces can completely obscure the tear. Systematic probing is necessary to demonstrate laminar instability and should be performed routinely, even when no surface abnormality is evident.

Within the avascular central disc, treatment consists of debridement to a stable rim; associated cysts are decompressed or excised during the same procedure [5, 60].

#### Delaminating (“flap”) tears and bucket-handle tears (NP-1B)

**Fig. 4:**
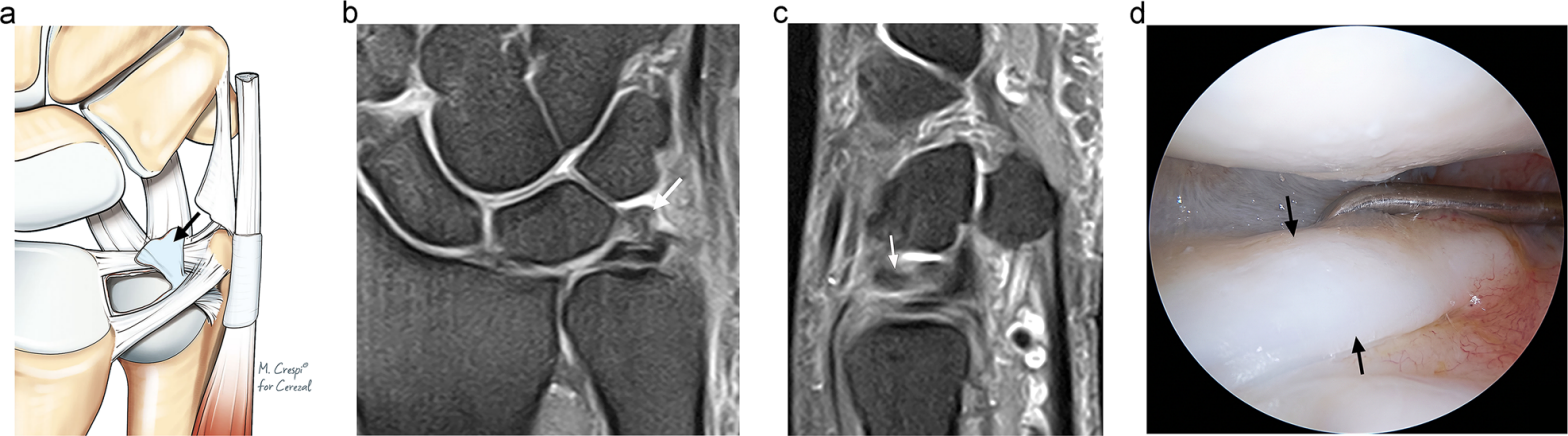
Communicating bucket-handle–type tear of the TFC proper (NP-1B). **a** Schematic drawing depicts delamination of the TFC proper with medial displacement of a TFC lamina (arrow). **b**, **c** Coronal and sagittal proton-density fat-suppressed MRI demonstrate a displaced flap in the ulnocarpal compartment, superficial to the ulnar TFCC insertion (arrows). **d** Arthroscopic image confirms the displaced TFC lamina attached medially by a pedicle (arrows)

**Fig. 5:**
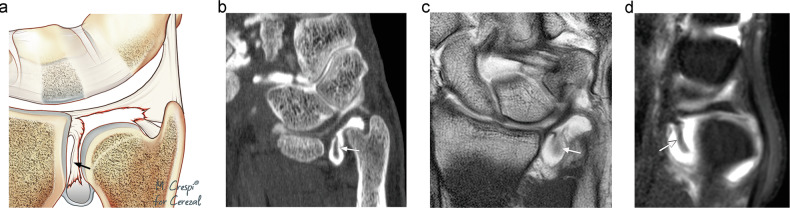
Proximal noncommunicating horizontal delaminating flap tear at the DRUJ margin (NP-1B). **a** Schematic drawing. **b** Coronal CT arthrography and (**c**, **d**) coronal T1-weighted and sagittal fat-suppressed T1-weighted MR arthrography demonstrate a proximally displaced horizontal flap within the DRUJ (arrows). Noncommunicating DRUJ-sided lesions may be missed at standard radiocarpal arthroscopy because the tear does not extend into the radiocarpal compartment

**Fig. 6:**
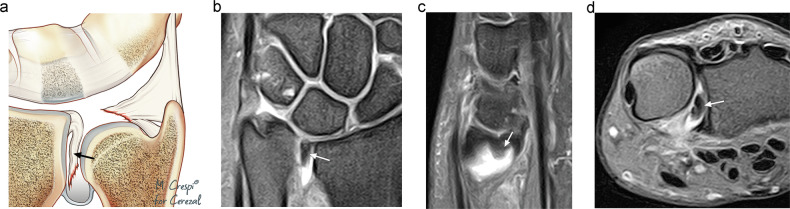
Distal communicating flap tear extending into the DRUJ (NP-1B). **a** Schematic drawing. **b**–**d** Coronal, sagittal, and axial proton-density fat-suppressed MRI demonstrate a central perforation of the TFC proper with a displaced fragment interposed within the DRUJ (arrows). This configuration may produce painful mechanical catching or locking with limited pronation–supination despite the absence of DRUJ instability

**Fig. 7:**
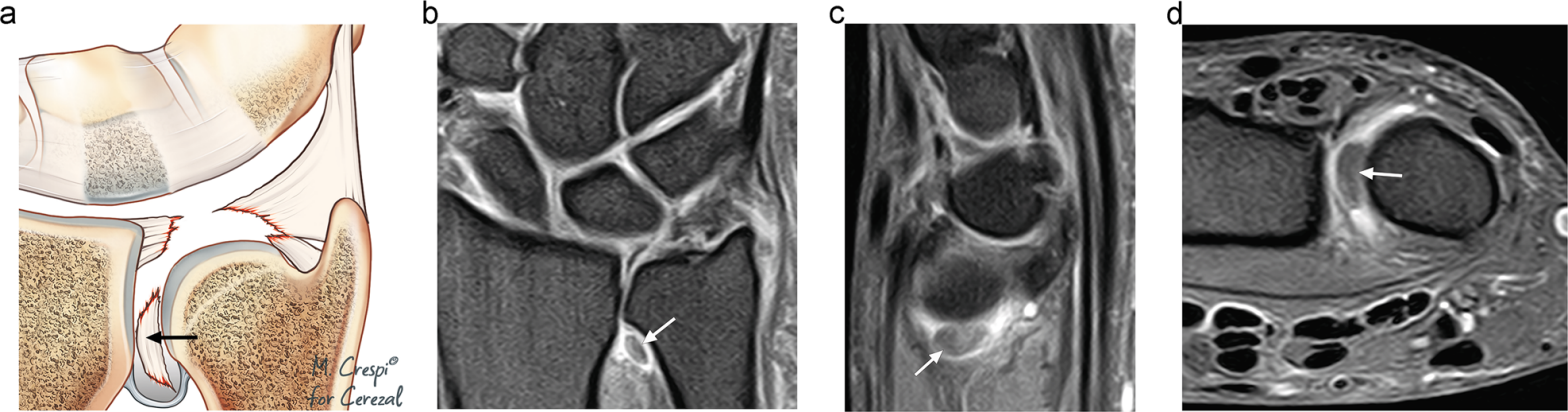
Central tear of the TFC proper with a free fragment in the proximal DRUJ recess (NP-1B). **a** Schematic drawing. **b**–**d** Coronal, sagittal, and axial proton-density fat-suppressed MRI reveal a complex tear of the TFC proper with a detached loose body within the proximal DRUJ recess (arrows)

Delaminating tears with unstable flaps and bucket-handle tears (NP-1B) are predominantly traumatic, although flaps may also arise from degenerative central perforations [21, 68]. In bucket-handle tears, one or more central TFC fragments fold back and displace into the radiocarpal compartment (Fig. 4), the DRUJ (Figs. 5 and 6), or both [68–72]. Fully detached fragments may migrate freely and produce symptoms similar to those of an intra-articular loose body (Fig. 7) [21, 71].

The typical patient is young, usually reports a history of trauma, and describes catching during loaded forearm rotation, sometimes with episodic locking [72, 73]. Bucket-handle displacement into the DRUJ is particularly important because the interposed fragment may become wedged between the ulnar head and the sigmoid notch, causing locking that does not resolve with simple manipulation [71].

On MRI, flap morphology, the presence or absence of compartment communication, and the fold-back configuration of bucket-handle tears, including displaced fragments within capsular recesses, can be identified [68–70]. The “comma sign,” consisting of a low-signal TFC fragment outlined by joint fluid, is the most characteristic finding on fluid-sensitive sequences [69]. Arthrography may improve the detection of noncommunicating and proximal flaps by distending the recesses in which they lie [21, 73].

Standard radiocarpal arthroscopy misses proximal flaps and DRUJ-displaced bucket-handle tears often enough that DRUJ portal evaluation should be planned preoperatively rather than considered only after an unrevealing radiocarpal assessment. Resection and debridement to a stable rim remain the standard treatment for lesions confined to the avascular central disc [73, 74].

### Capsular detachments of the TFCC (NP-2)

Capsular TFCC lesions (NP-2) remain underrecognized on imaging, largely because most musculoskeletal radiologists are far more accustomed to the classic Palmer tear patterns. Del Piñal first systematized these injuries into four groups: volar capsular detachment, dorsal capsular detachment, ulnocarpal ligament injuries, and carpal detachment, the so-called Nishikawa lesion [5]. Beyond these four groups, two additional entities belong to the spectrum of NP capsular injuries: triquetral impingement ligament tear (TILT syndrome), regarded as the chronic counterpart of the Nishikawa lesion [5], and proximal avulsion of the radiocarpal capsule (PARC) [75]. Because PARC involves the dorsal capsular region, it is discussed together with dorsal capsular detachment in the corresponding section.

#### Volar capsular detachment (NP-2A)

**Fig. 8:**
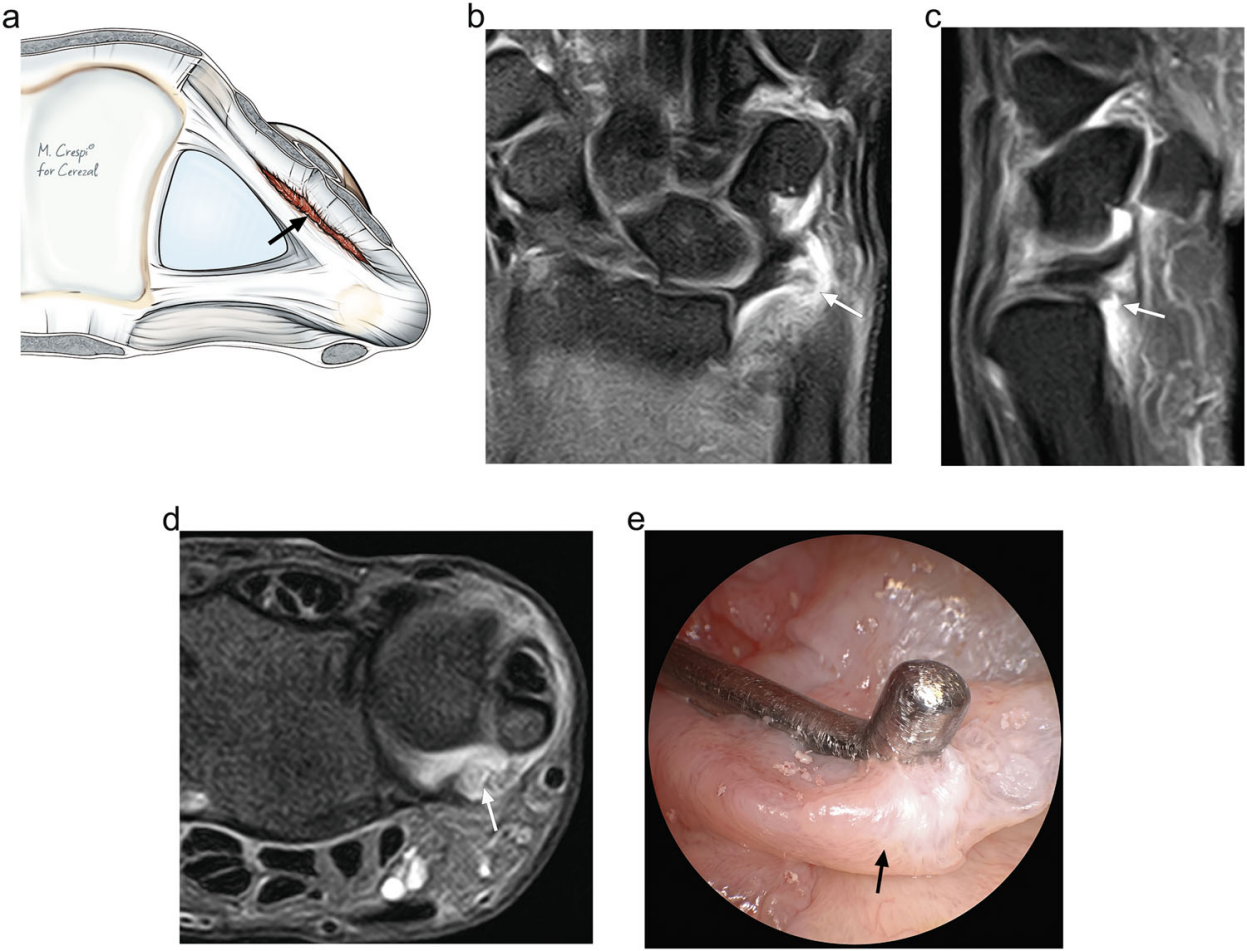
Volar capsular detachment of the TFCC (NP-2A). **a** Axial schematic drawing depicts a volar capsular tear involving the capsular insertion of the volar DRUL (arrow). **b**–**d** Coronal, sagittal, and axial proton-density fat-suppressed MRI demonstrate disruption of the volar capsular attachment (arrows). **e** Arthroscopic image confirms detachment of the volar DRUL from the volar capsule, with anterior folding of the TFCC dorsally (arrow)

Only isolated cases of volar capsular detachment (NP-2A) have been described. The lesion consists of separation of the volar-ulnar TFCC margin, including the volar DRUL component, from the palmar capsule (Fig. 8) [5]. Patients typically present with nonspecific volar-ulnar wrist pain without DRUJ instability [5].

On sagittal and axial MRI, the key finding is a fluid-signal cleft between the volar TFCC margin and the palmar capsule, sometimes accompanied by focal synovitis or pericapsular edema [21]. MR or CT arthrography may improve detection, as capsular distension more clearly delineates the detachment plane. Arthroscopy confirms the diagnosis; repair is performed when tissue quality permits, and debridement when it does not [5].

#### Dorsal capsular detachment (NP-2B)

**Fig. 9:**
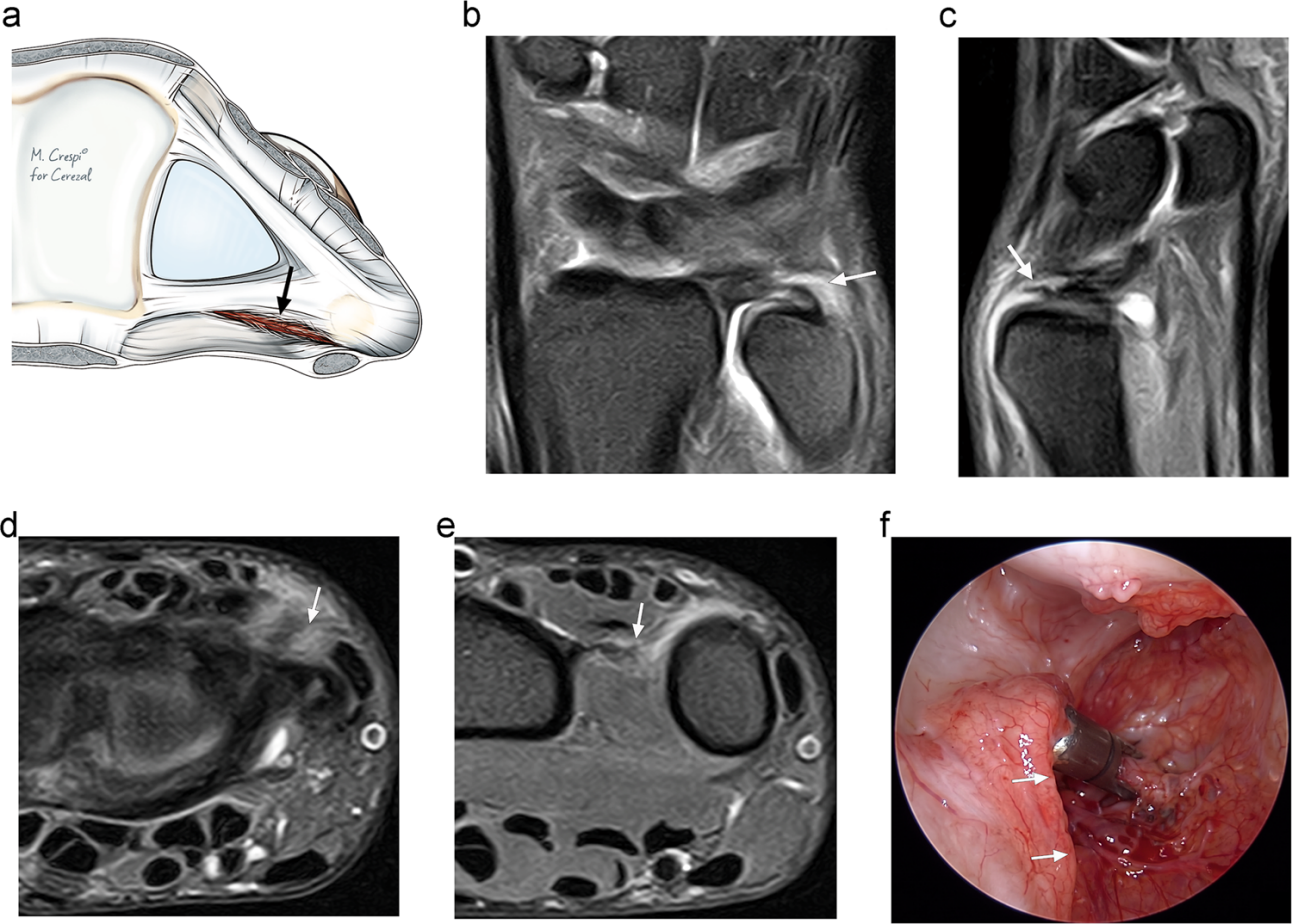
Dorsal capsular detachment of the TFCC (NP-2B). **a** Axial schematic drawing depicts a dorsal capsular tear involving the capsular attachment of the dorsal DRUL (arrow). **b**–**d** Coronal, sagittal, and axial proton-density fat-suppressed MRI demonstrate disruption of the dorsal capsular attachment with focal synovitis and pericapsular edema (arrows). **e** Axial proton-density fat-suppressed MRI reveals associated involvement of the distal radioulnar band of the distal interosseous membrane (arrow). **f** Arthroscopic image confirms detachment of the dorsal DRUL from the dorsal capsule with focal synovitis (arrows)

Dorsal capsular detachment (NP-2B) is among the most frequently identified NP injury patterns and a persistently underdiagnosed cause of ulnar-sided wrist pain, particularly in athletes who load the wrist in rotation [5, 76, 77]. The injury involves separation of the dorsal TFCC disc margin, the dorsal DRUL component, or both from the dorsal capsule (Fig. 9) [5, 76, 77]. This lesion is anatomically related to the distal radioulnar tract of the interosseous membrane, which inserts into the dorsal capsule between the fifth and sixth extensor compartments and extends toward the foveal TFCC attachment [78]. When a dorsal capsular detachment is present, concurrent ECU subsheath injury and foveal tears should be suspected, as the combination may produce DRUJ instability disproportionate to the severity of each isolated lesion [21, 79].

Patients report dorsoulnar pain with forearm rotation and ulnocarpal stress, typically related to torsional loading in racquet or throwing sports. Isolated lesions are usually mechanically stable. A positive DRUJ ballottement test points toward a concomitant foveal injury [76, 77].

On MR imaging, dorsal capsular detachment appears as a fluid-signal cleft between the dorsal TFCC margin and the dorsal capsule, usually accompanied by focal synovitis [76, 80]. MR or CT arthrography distends the dorsal recess and improves delineation of the detachment plane [21]. Arthroscopy confirms the tear and guides repair; the peripheral vascularity of this zone favors healing [81, 82].

PARC affects the same dorsal capsular region. PARC results from blunt wrist trauma or sudden blocking of palmar flexion, as in a tennis forehand or a blocked golf swing, and consists of traumatic detachment of the proximal dorsal radiocarpal capsule from the dorsal radial rim, with variable extension ulnarly into the dorsal TFCC or radially into the dorsal capsuloligamentous scapholunate septum [75]. The foveal insertion is typically intact, and the DRUJ is stable in most cases [75]. The lesion is frequently hidden by overlying fibrous tissue and may go undetected at arthroscopy unless systematic debridement of the dorsal capsular insertion is performed [75]. Treatment consists of arthroscopic suture repair [75].

#### Injuries of the volar ulnocarpal ligaments (NP-2C)

Volar ulnocarpal ligament injuries (NP-2C) include Palmer type 1 C distal avulsions of the UL and/or UT ligaments [1], and the less-recognized longitudinal intraligamentous split of the UT [83]. The UT originates from the palmar radioulnar ligament and inserts onto the triquetrum [26]. A structurally weak zone at the prestyloid recess and pisotriquetral aperture predisposes to longitudinal cleavage rather than transverse avulsion, a distinction with direct implications for repair strategy [83].

The usual mechanism is wrist hyperextension, forearm supination, and axial loading. Most patients present with chronic ulnar wrist pain and no DRUJ instability [83].

MRI has poor sensitivity for longitudinal UT splitting [84]. Intraligamentous signal changes, when present, are often nonspecific, and MRI is most useful in this setting for excluding other diagnoses [84]. Arthroscopy remains the reference standard for confirming the tear. Preliminary synovial debridement may be needed to expose the cleavage plane; once visible, outside-in suture repair has produced good functional results in reported series [85].

#### Nishikawa lesion-carpal detachment (NP-2D)

**Fig. 10:**
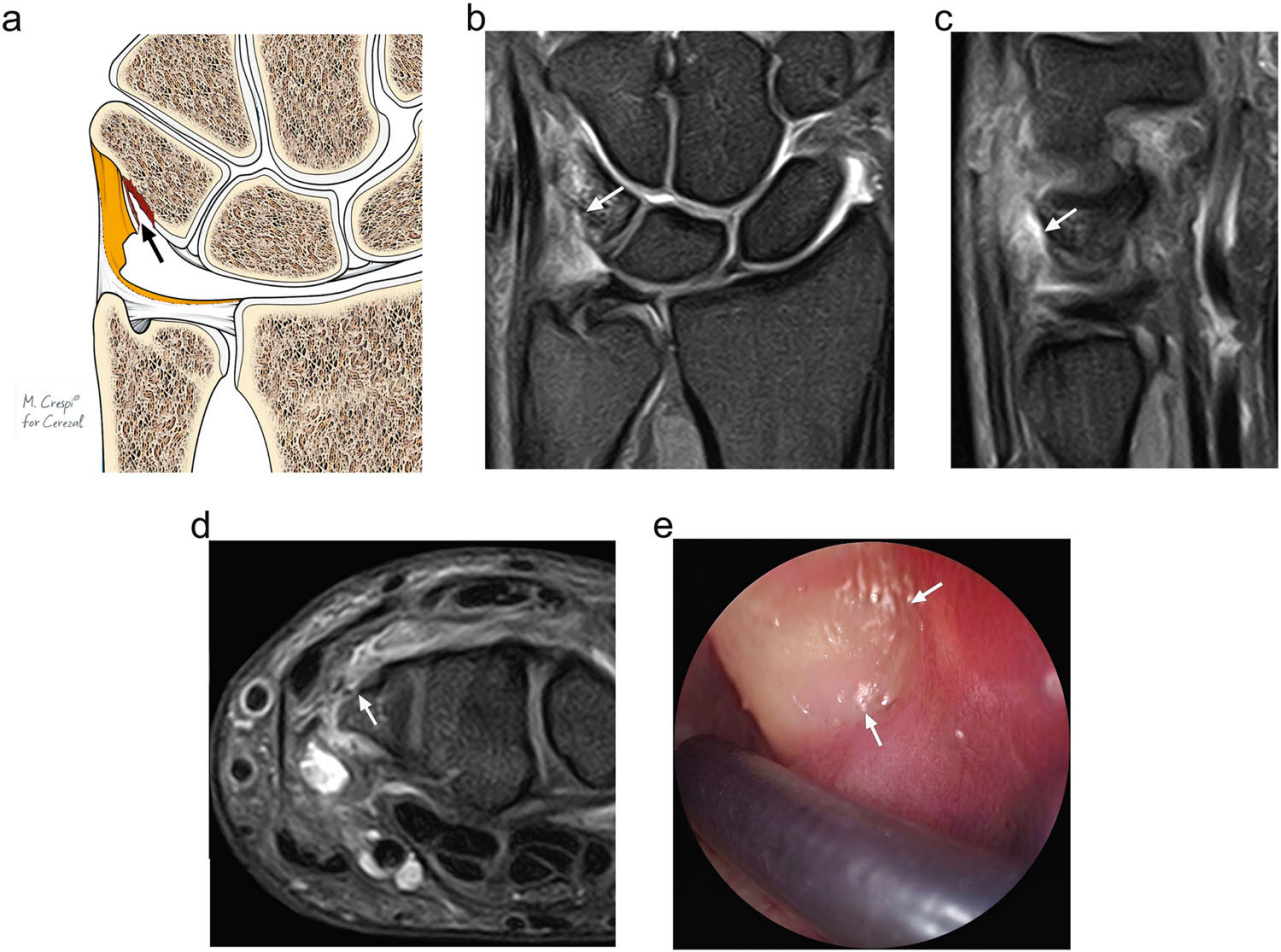
Ulnocarpal detachment or Nishikawa lesion (NP-2D). **a** Coronal schematic drawing illustrates avulsion of the dorsoulnar ulnocarpal capsule and meniscus homologue (UMH) from the dorsal triquetrum, with focal chondral delamination (arrow) and synovitis. **b**–**d** Coronal, sagittal, and axial proton-density fat-suppressed MRI demonstrate detachment of the dorsal triquetral insertion of the dorsoulnar ulnocarpal capsule and UMH (arrows), with focal synovitis, fibrosis, chondral delamination, and bone marrow edema at the dorsal triquetral margin. **e** Arthroscopic image reveals focal avulsive chondral delamination at the dorsal triquetrum (arrows) with adjacent synovitis

The Nishikawa lesion (NP-2D) is a detachment of the distal capsulomeniscal complex from the dorsoulnar triquetral margin [86]. The avulsion may involve the dorsoulnar ulnocarpal capsule, the dorsal radiocarpal ligament, and the UMH [5, 86]. Mobile residual tissue within the dorsoulnar recess perpetuates synovitis and, through chronic abrasion, produces the characteristic dorsal triquetral chondral defect (Fig. 10) [5, 86]. A covered-type UMH insertion with broad dorsal triquetral coverage has been proposed as a predisposing anatomic variant [12].

Mechanisms include a fall onto a supinated hand, torsional trauma, hyperflexion, or repetitive loading. Patients typically report persistent dorsoulnar pain after what is initially presumed to be a simple sprain, with symptoms worsening during forearm rotation and ulnar deviation, usually without DRUJ instability. This nonspecific presentation often delays diagnosis [5, 86].

MRI is the first imaging step. The key findings are focal dorsoulnar synovitis or fibrosis and a dorsal triquetral chondral defect with reactive subchondral changes, best assessed on sagittal and axial planes [8, 15, 80]. MR or CT arthrography more clearly delineates the detachment plane and may reveal mobile UMH tissue within the dorsoulnar recess [50]. At arthroscopy, mobile tissue and focal synovitis in the dorsoulnar recess are the hallmark findings. Treatment consists of synovectomy with resection of unstable tissue; reported outcomes have been favorable [5, 86].

#### TILT syndrome-TILT (NP-2E)

**Fig. 11:**
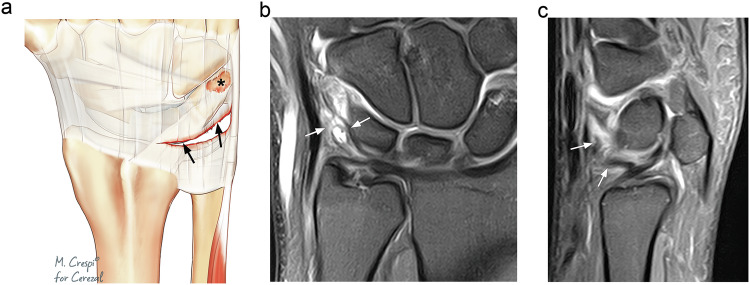
TILT syndrome (NP-2E). **a** Schematic drawing illustrates proximal avulsion of a fibrous cuff from the ulnar sling mechanism (arrows), with distal migration and repetitive impingement against the ulnar slope of the triquetrum (asterisk). Focal synovitis, progressive chondral damage, and reactive subchondral changes may result. **b**, **c** Coronal and sagittal proton-density fat-suppressed MRI demonstrate disruption of the ulnar sling mechanism (arrows) with focal synovitis, chondropathy, and subchondral changes at the dorsoulnar triquetrum

TILT syndrome (NP-2E) results from chronic impingement caused by proximal detachment of a fibrous cuff from the ulnar sling mechanism, the continuous ring of soft tissue ulnar to the radius that includes the extensor retinaculum, dorsal radioulnar ligament, ECU subsheath, ulnocarpal ligaments, and distal TFCC (Fig. 11) [87]. The displaced cuff migrates distally and impinges against the ulnar slope of the triquetrum, producing localized hyperemia and progressive cartilage loss, with eventual cortical softening [87]. Prior ulnar-sided surgery that disrupts sling integrity may predispose to the same mechanism [87]. The Nishikawa lesion is an acute distal capsulomeniscal avulsion; TILT is its chronic counterpart, distinguished by secondary osseous remodeling. A pathophysiologic continuum between both entities has been proposed [87].

Clinical diagnosis rests on Watson’s triad: a hyperflexion or direct-trauma event, focal dorsoulnar triquetral tenderness, and normal radiographs [87]. MRI demonstrates fibrotic or synovial tissue in the dorsoulnar ulnocarpal compartment, dorsal triquetral chondropathy, marrow edema, and occasional reactive sclerosis or subchondral cysts [8, 80]. MR or CT arthrography may improve delineation of the dorsal capsular recess [8]. Initial management is conservative; refractory cases are treated with arthroscopic resection of the fibrous cuff and chondroplasty [87].

### Complex or combined TFCC injuries (NP-3)

**Fig. 12:**
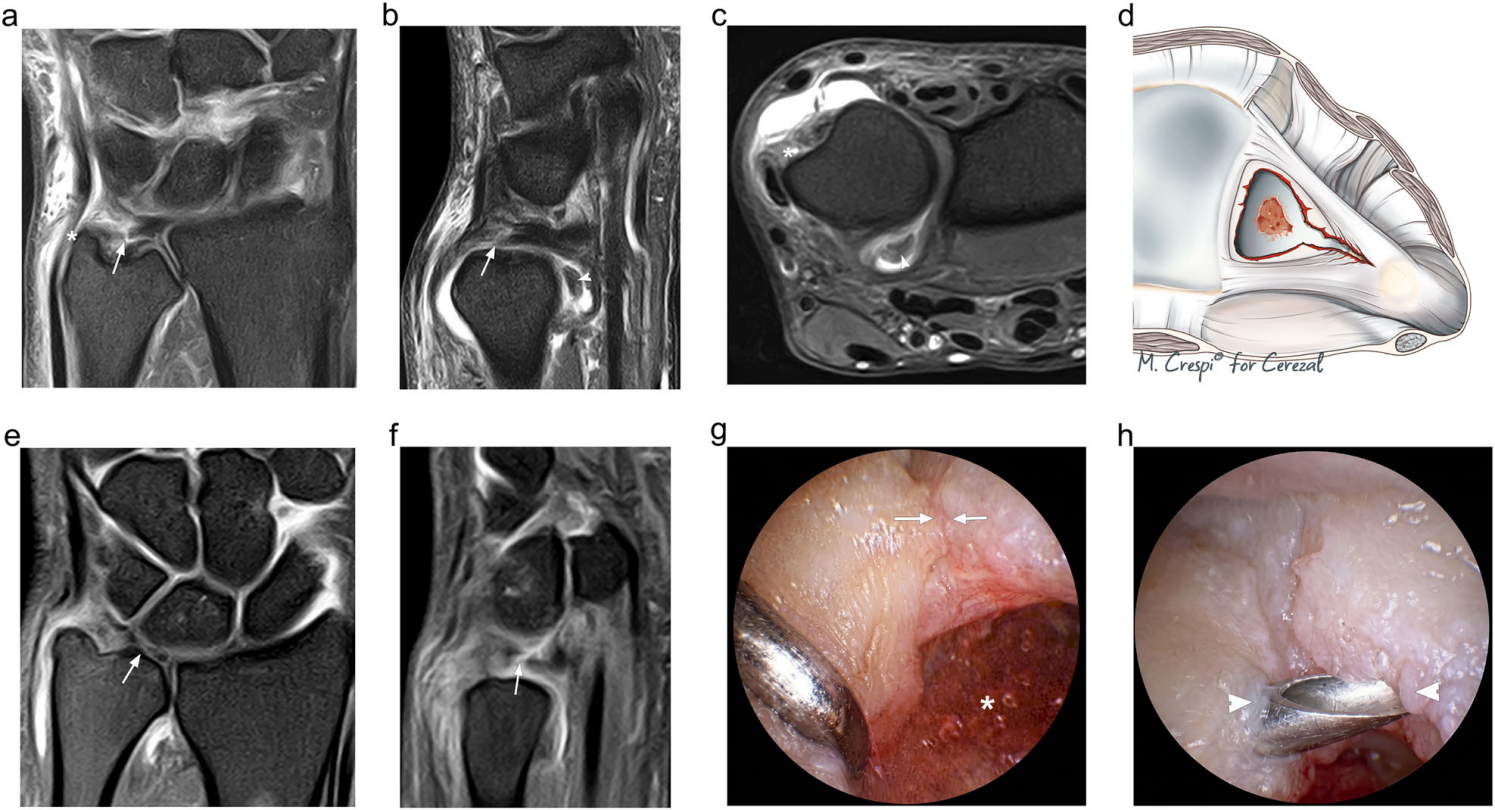
Complex (combined) NP TFCC injuries (NP-3). **a**–**c** Coronal, sagittal, and axial proton-density fat-suppressed MRI demonstrate a complete tear of the ulnar TFCC attachment (Palmer 1B) (arrows), with a detached central TFC fragment displaced into the DRUJ (arrowheads). An associated ECU subsheath tear is also present (asterisks), with ECU subluxation and tenosynovitis, representing a combined traumatic pattern (NP-3A). **d** Schematic drawing illustrates a coronal TFCC tear combining a degenerative lesion (Palmer 2 C) with a traumatic ulnar-sided coronal longitudinal split that separates the dorsal and volar DRULs at their ulnar attachments. **e**, **f** Coronal and sagittal proton-density fat-suppressed MRI depict the same mixed traumatic-degenerative pattern, with a central degenerative perforation (arrow in e) and associated coronal splitting of the ulnar attachment (arrow in **f**) (NP-3B). **g**, **h** Arthroscopic images show a central perforation treated with an arthroscopic wafer procedure (asterisk) and an ulnar-sided coronal tear (arrows) repaired with suture of the ulnar DRUL (arrowheads). Note: all original schematic illustrations in this article were created by Maximiliano Crespi, commissioned by Dr. Cerezal for this article, and are used with permission

Complex TFCC injuries (NP-3) fall into two groups: combined traumatic lesions and mixed traumatic-degenerative lesions in which acute injury is superimposed on a previously degenerated disc (Fig. 12). Combined traumatic patterns account for approximately 18–22% of traumatic TFCC lesions in arthroscopy-correlated series [46, 60].

#### Combined traumatic patterns (NP-3A)

Combined traumatic lesions (NP-3A) follow either a single high-energy event or repetitive loading and may present in several configurations. The most common pairing is central disc perforation with a peripheral ulnar-sided tear (Palmer 1 A + 1B). These two components require different treatments: central debridement for the disc tear and peripheral repair if instability is confirmed. Failure to identify both components preoperatively may lead to incomplete treatment [45, 46, 88].

A second clinically important configuration combines peripheral ulnar-sided tearing with dorsal capsular detachment and foveal involvement. The foveal component is the critical determinant of instability, and its disruption substantially increases the risk of DRUJ instability even when each individual tear appears modest in isolation [20, 21].

At the far end of the severity spectrum, simultaneous radial and ulnar detachment leaves the TFCC without bony constraint on either side, a configuration termed the floating TFCC [89]. Instability in this setting occurs in both the sagittal and coronal planes, and repair of both sides is usually required [89].

#### Mixed traumatic-degenerative patterns (NP-3B)

Mixed lesions (NP-3B) develop when an acute injury occurs in a degenerated disc [8, 21]. The coronal tear is a typical example: a pre-existing central perforation extends coronally toward the fovea, potentially splitting the ulnar attachment of both the volar and dorsal DRUL. What began as a degenerative central defect can thus acquire the instability profile of a foveal avulsion [90].

These configurations share a practical problem: conventional MRI underestimates them. Foveal extension and proximal disease in particular are difficult to characterize without arthrographic distension. When foveal extension or proximal disease is suspected, whether on the basis of a positive ballottement test or because the imaging picture appears incomplete, MR or CT arthrography should be performed to map the full extent of the lesion before surgery [8, 50].

During arthroscopy, all involved compartments need to be assessed. If preoperative imaging raises the possibility of proximal disease, DRUJ portal inspection is not optional. These combined patterns carry a substantially higher instability risk than isolated tears, and if surgical planning addresses only the most visible component, the primary source of instability may remain untreated [9, 57].

## Conclusion

The Palmer classification remains the main framework for assessing TFCC injuries. More than three decades after its introduction, it remains practical, reproducible, and widely understood in both radiologic and surgical practice. Subsequent advances in arthroscopy and imaging, however, have shown that the original system did not encompass the full spectrum of TFCC pathology, an expected limitation given the diagnostic perspective available at the time of its development.

The refinements of types 1B and 1D have addressed some of the most clinically important gaps. The NP patterns reviewed here, including central delaminating lesions, capsular detachments, and complex combined configurations, account for additional injury patterns with clear diagnostic and therapeutic relevance. These lesions share two clinically important features: they are frequently overlooked on conventional assessment, and failure to recognize them may lead to incomplete surgical planning.

A topographic framework based on anatomic location is not intended to replace the Palmer classification, but to complement it. Accurate identification of these NP patterns is essential for appropriate treatment planning in patients with ulnar-sided wrist pain.

## Electronic supplementary material

Supplementary information

## Data Availability

Data sharing is not applicable to this article, as no datasets were generated or analysed during the current study. All illustrative cases derive from the authors’ clinical practice.

## Abbreviations

DRUJ: Distal radioulnar joint
DRUL: Distal radioulnar ligament
ECU: Extensor carpi ulnaris
NP: Non-Palmer
PARC: Proximal avulsion of the radiocarpal capsule
TFC: Triangular fibrocartilage
TFCC: Triangular fibrocartilage complex
TILT: Triquetral impingement ligament tear
UC: Ulnocapitate ligament
UL: Ulnolunate ligament
UMH: Ulnomeniscal homologue
UT: Ulnotriquetral ligament
