# Supplementary material for: Beyond Palmer: a topographic classification of non-Palmer triangular fibrocartilage complex injuries
Source: Insights Imaging. 2026 Aug 6;17:199. doi: 10.1186/s13244-026-02356-8 (PMC13447642; doi:10.1186/s13244-026-02356-8)

Beyond Palmer: A Topographic Classification of Non-Palmer Triangular Fibrocartilage Complex Injuries

Electronic supplementary material

Supplementary material

Fig. S1 Diagnostic and management algorithm for non-Palmer TFCC lesions. After lesion characterization with MRI, MR arthrography, or CT arthrography, non-Palmer lesions are classified as central (NP-1), capsular (NP-2), or combined (NP-3) patterns, each with distinct arthroscopic treatment implications.

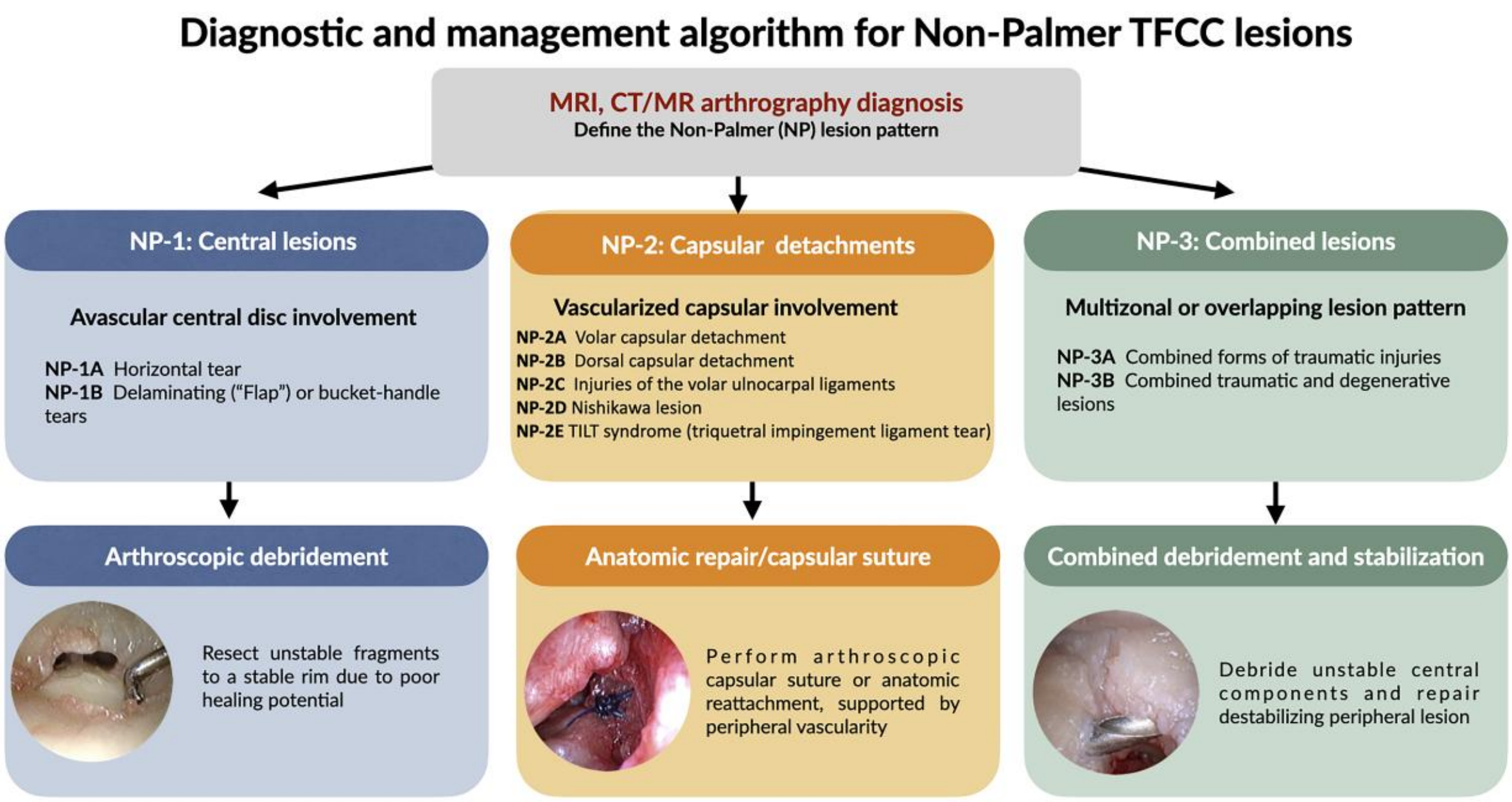

Supplement: Supplementary file 1 [file 13244_2026_2356_MOESM1_ESM.pdf]
